# Supplementary material for: Identification of Antidiabetic Compounds from the Aqueous Extract of Sclerocarya birrea Leaves
Source: Molecules. 2022 Nov 21;27(22):8095. doi: 10.3390/molecules27228095 (PMC9696724; doi:10.3390/molecules27228095)
Supplement: Supplementary file 1 [file molecules-27-08095-s001.zip › molecules-1996023-supplementary.pdf]

Supporting Information (SI) for:

Bioassay-guided isolation of bioactive compounds from the leaf extract of *Sclerocarya birrea* and their glucose uptake potential in skeletal myocytes

Table of contents

(S1) ^{1}H NMR and ^{13}C NMR data of Myricetin (**1**) in methanol-*d4* compared to those reported by (He et al.,2009) in DMSO-*d6*

(S2) ^{1}H NMR and ^{13}C NMR data of Myricetin-3-O-β-D-glucuronide (**2**) in methanol-*d4* compared to those reported by (Granica et al., 2013) in methanol-*d4*

(S3) ^{1}H NMR and ^{13}C NMR data of Quercetin-3-O-β-D-glucuronide (**3**) in methanol-*d4* compared to those reported by (Moon et al., 2001) in methanol-*d4*

(S4) Relative Glucose uptake activity of Marula fractions in C2C12 myocytes over a range of 0.01-100μg/ml. Activity is expressed relative % to the baseline glucose uptake (control) set at 0% and the positive control insulin (Ins) set at 100%. Active fraction (fraction 3) exhibited comparable potency to Insulin. P value < *p < 0.05, **p < 0.01. ***p < 0.001

(S5) HRESIMS spectrum of Myricetin (**1**)

(S6) HRESIMS spectrum of Myricetin-3-O-β-D-glucuronide (**2**)

(S7) HRESIMS spectrum of Quercetin-3-O-β-D-glucuronide (**3**)

(S8) MS fragmentation pattern of peak 1 overlaid with MSMS fragmentation pattern of peak 1

(S9) MS fragmentation pattern of peak 2 overlaid with MSMS fragmentation pattern of peak 2

(S10) MS fragmentation pattern of peak 3 overlaid with MSMS fragmentation pattern of peak 3

(S11) MS fragmentation pattern of peak 4 overlaid with MSMS fragmentation pattern of peak 4

(S12) MS fragmentation pattern of peak 5 overlaid with MSMS fragmentation pattern of peak 5

(S13) MS fragmentation pattern of peak 6 overlaid with MSMS fragmentation pattern of peak 6

Table S1 ^{1}H NMR and ^{13}C NMR data of Myricetin (**1**) in methanol-*d4* compared to those reported by (He et al.,2009) in DMSO-*d6*

Position	^{1}H (ppm, J in Hz) of isolated myricetin in CD_3OD	^{1}H (ppm, J in Hz) literature data in DMSO-*d6*	^{13}C (ppm) of isolated myricetin in CD_3OD	^{13}C (ppm, J in Hz) literature data in DMSO-*d6*
2			145.5	146.8
3			136.5	135.9
4			178.3	175.7
5			161.8	160.7
6	6.22 (1H, d, J=2.09 Hz)	6.18 (1H, d, J=2.40 Hz)	98.4	98.2
7			164.5	164.1
8	6.39 (1H, d, J=2.17 Hz)	6.37 (1H, d, J=1.8 Hz)	93.3	93.2
9			157.1	156.1
10			102.2	102.9
1’			120.5	120.7
2’6’	6.97 (2H, s)	7.24 (2H, s)	108.1	107.1
3’			145.5	145.7
4’			134.9	135.8
5’			145.5	145.7

Table S2 ^{1}H NMR and ^{13}C NMR data of Myricetin-3-O-β-D-glucuronide (**2**) in methanol-*d4* compared to those reported by (Granica et al., 2013) in methanol-*d4*

Position	^{1}H (ppm, J in Hz) of isolated myricetin-3-O-β-D-glucuronide in CD_3OD	^{1}H (ppm, J in Hz) literature data in CD_3OD	^{13}C (ppm) of isolated myricetin-3-O-β-D-glucuronide in CD_3OD	^{13}C (ppm, J in Hz) literature data in CD_3OD
2			157.5	156.2
3			134.1	133.2
4			177.8	177.1
5			161.7	161.2
6	6.21(1H, d, J=2.05 Hz)	6.20 (1H, d, J=1.90 Hz)	98.5	98.7
7			164.6	164.2
8	6.39 (1H, d, J=2.00 Hz)	6.38 (1H, d, J=1.90 Hz)	93.2	93.4
9			157.0	156.2
10			102.7	101.1
1’			120.3	119.7
2’	7.29 (2H, s)	7.28 (2H, s)	108.5	108.5
3’			145.0	145.4
4’			136.7	136.8
5’			145.0	145.4
6’	7.29 (2H, s)	7.28 (2H, s)	108.5	108.5
1’’	5.39 (1H, d, J=7.88Hz)	5.36 (1H, d, J=7.60 Hz)	104.2	103.8
2’’	3.49-3.61 (3H, m)	3.66-3.43 (3H, m)	73.9	73.6
3’’	3.49-3.61 (3H, m)	3.66-3.43 (3H, m)	76.3	76.0
4’’	3.49-3.61 (3H, m)	3.66-3.43 (3H, m)	71.6	71.2
5’’	3.75 (1H, d, J=9.68 Hz)	3.77 (1H, d, J=9.50 Hz)	75.8	75.9
6’’			171.7	169.8

Table S3 ^{1}H NMR and ^{13}C NMR data of Quercetin-3-O-β-D-glucuronide (**3**) in methanol-*d4* compared to those reported by (Moon et al., 2001) in methanol-*d4*

Position	^{1}H (ppm, J in Hz) of isolated quercetin-3-O-β-D-glucuronide in CD_3OD	^{1}H (ppm, J in Hz) literature data in CD_3OD	^{13}C (ppm) of isolated quercetin-3-O-β-D-glucuronide in CD_3OD	^{13}C (ppm, J in Hz) literature data in CD_3OD
2			161.6	159.2
3			134.5	135.5
4			177.9	179.4
5			164.7	163.2
6	6.10 (1H, d, J= 2.05Hz)	6.21 (1H, d, J= 2.00Hz)	101.6	100.1
7			168.2	166.2
8	6.30 (1H, d, J= 2.05Hz)	6.40 (1H, d, J= 2.00Hz)	93.3	94.9
9			157.7	158.6
10			104.26	105.8
1’			125.8	123.0
2’	7.78 (1H, bs)	7.60-7.67 (2H, m)	114.7	116.2
3’			144.5	146.1
4’			148.5	150.0
5’	6.75 (1H, d, J= 8.48Hz)	6.85 (1H, d, J= 8.48Hz)	116.4	117.4
6’	7.44 (1H, dd)	7.60-7.67 2H, m)	121.5	123.6
1’’	5.32 (1H, d, J= 7.57Hz	5.34 (1H, d, J= 7.40Hz	103.9	104.4
2’’	3.38-3.49(3H, m)	3.42-3.66 (3H, m)	74.1	75.5
3’’	3.38-3.49	3.42-3.66 (3H, m)	76.6	77.7

4’’	3.38-3.49	3.42-3.66 (3H, m)	73.7	73.0
5’’	3.55 (1H, d, J= 9.7Hz	3.75 (1H, d, J= 9.60 Hz	76.5	77.2
6’’			174.9	172.4

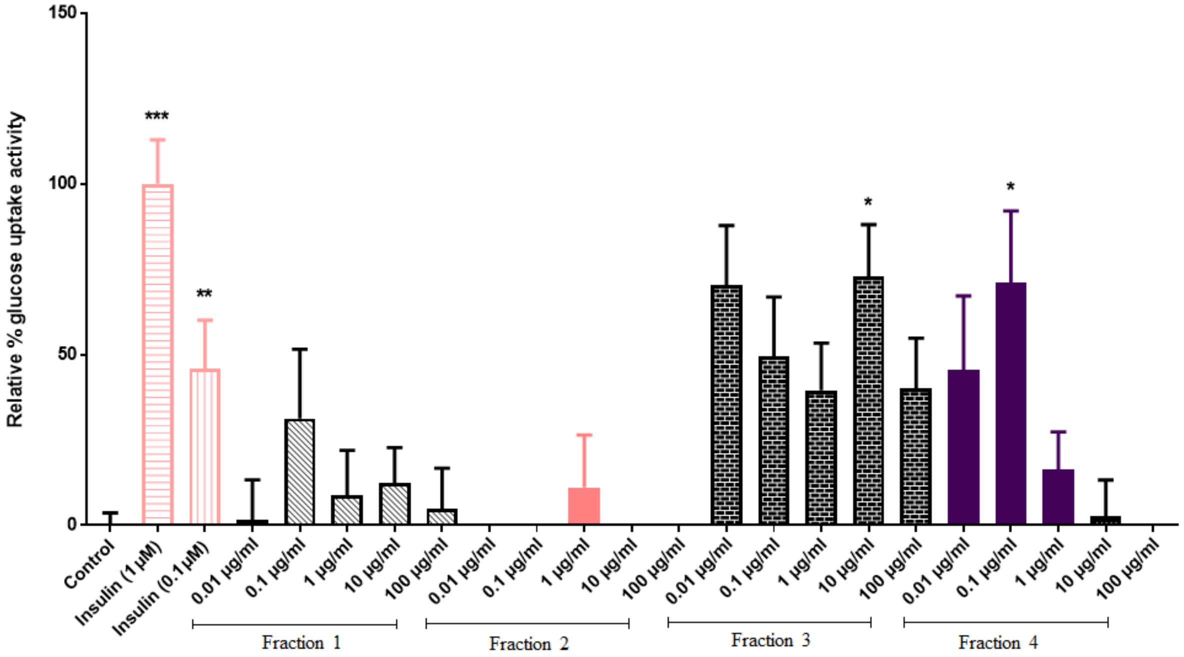

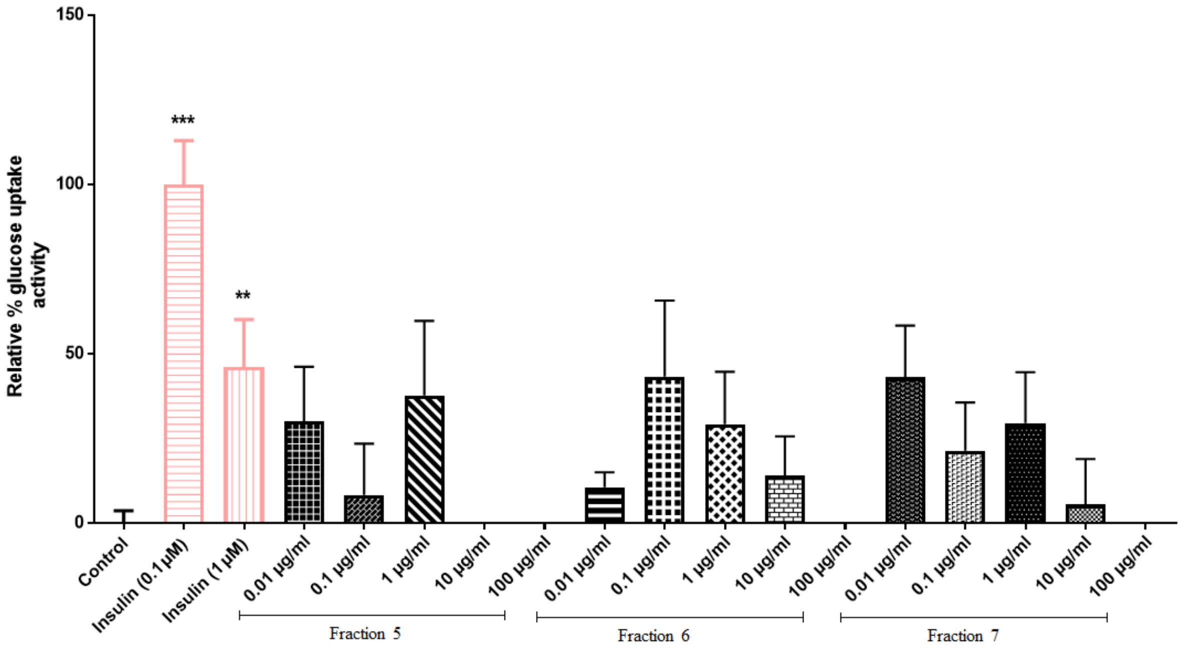

Fig. S4. Relative Glucose uptake activity of Marula fractions in C2C12 myocytes over a range of 0.01-100µg/ml. Activity is expressed relative % to the baseline glucose uptake (control) set at 0% and the positive control insulin (Ins) set at 100%. Active fraction (fraction 3) exhibited comparable potency to Insulin. P value < *p < 0.05, **p < 0.01. ***p < 0.001

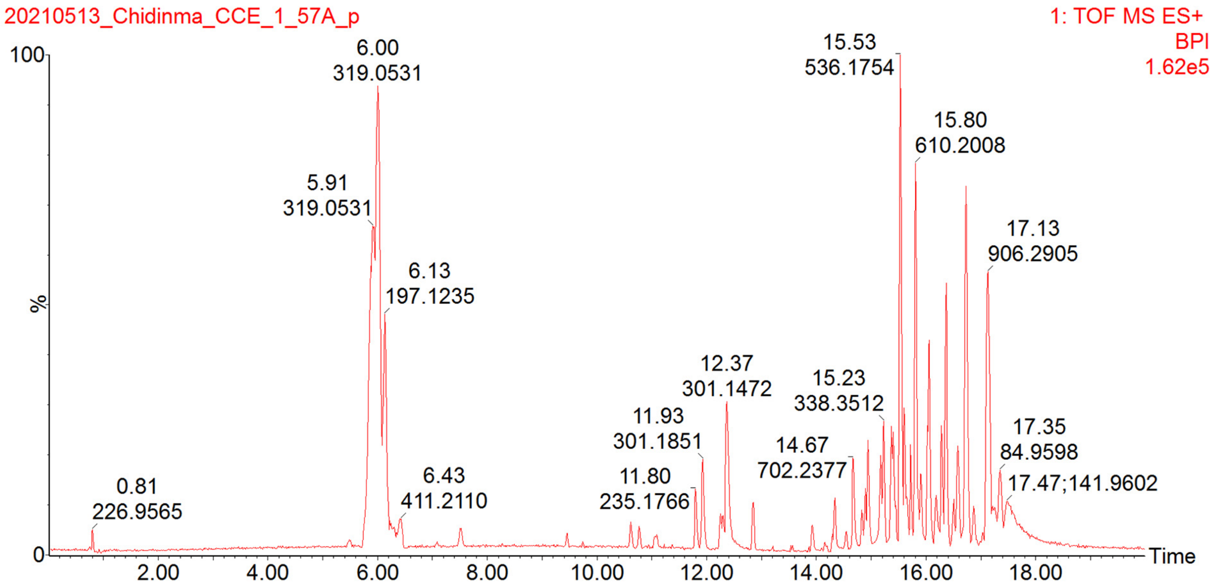

Fig. S5. ESI negative-mode BPI chromatogram of compound **1** (Myricetin) isolated from Fraction 4

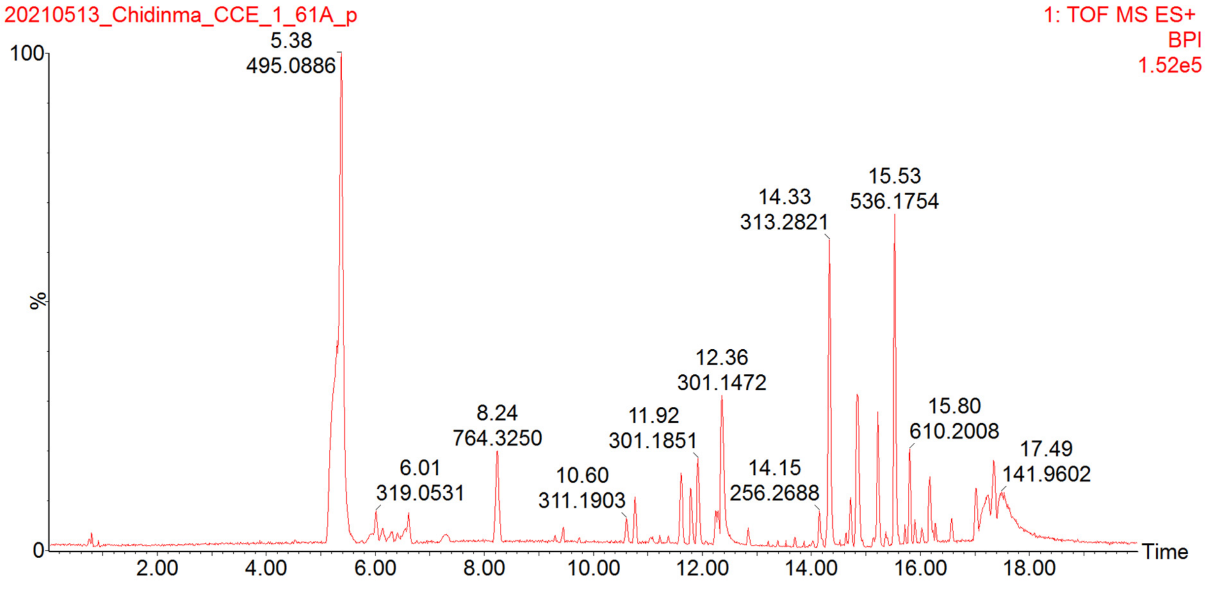

Fig. S6. ESI negative-mode BPI chromatogram of compound **2** (Myricetin-3-O-β-D-glucuronide) isolated from Fraction 3

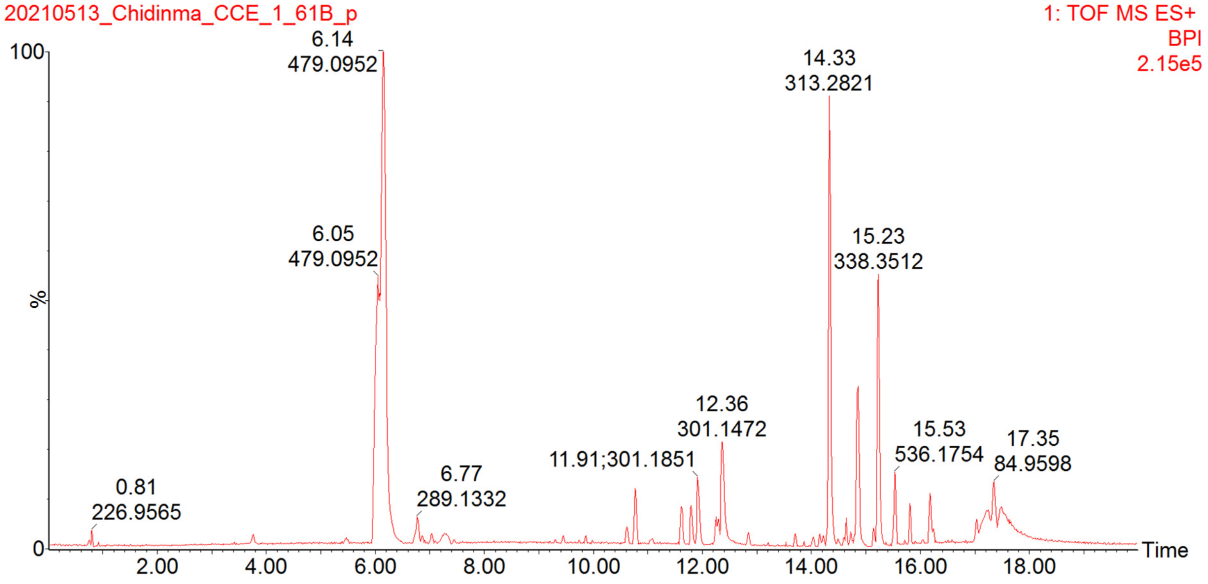

Fig. S7. ESI negative-mode BPI chromatogram of compound **3** **(**Quercetin-3-O-β-D-glucuronide) isolated from Fraction 3

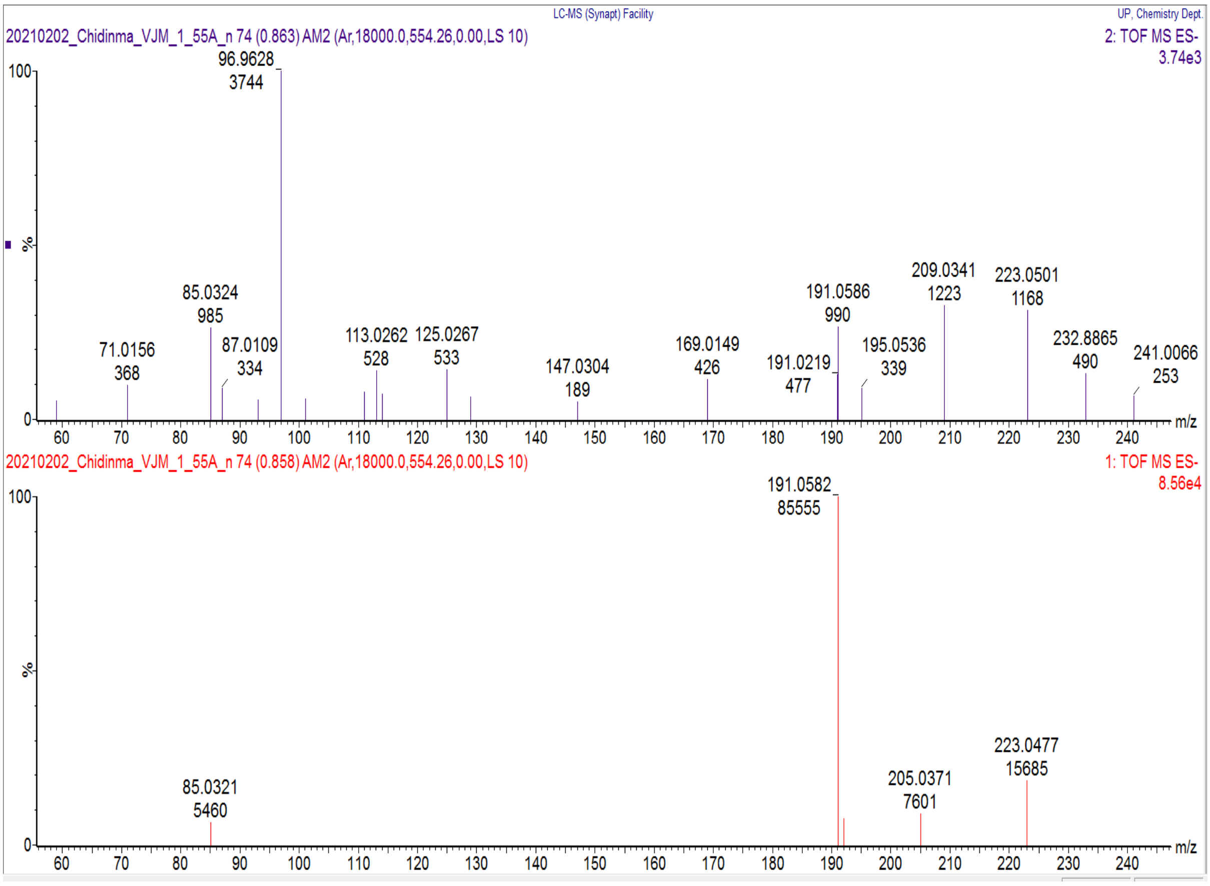

Fig.S8. MS fragmentation pattern of peak 1 overlaid with MSMS fragmentation pattern of peak 1

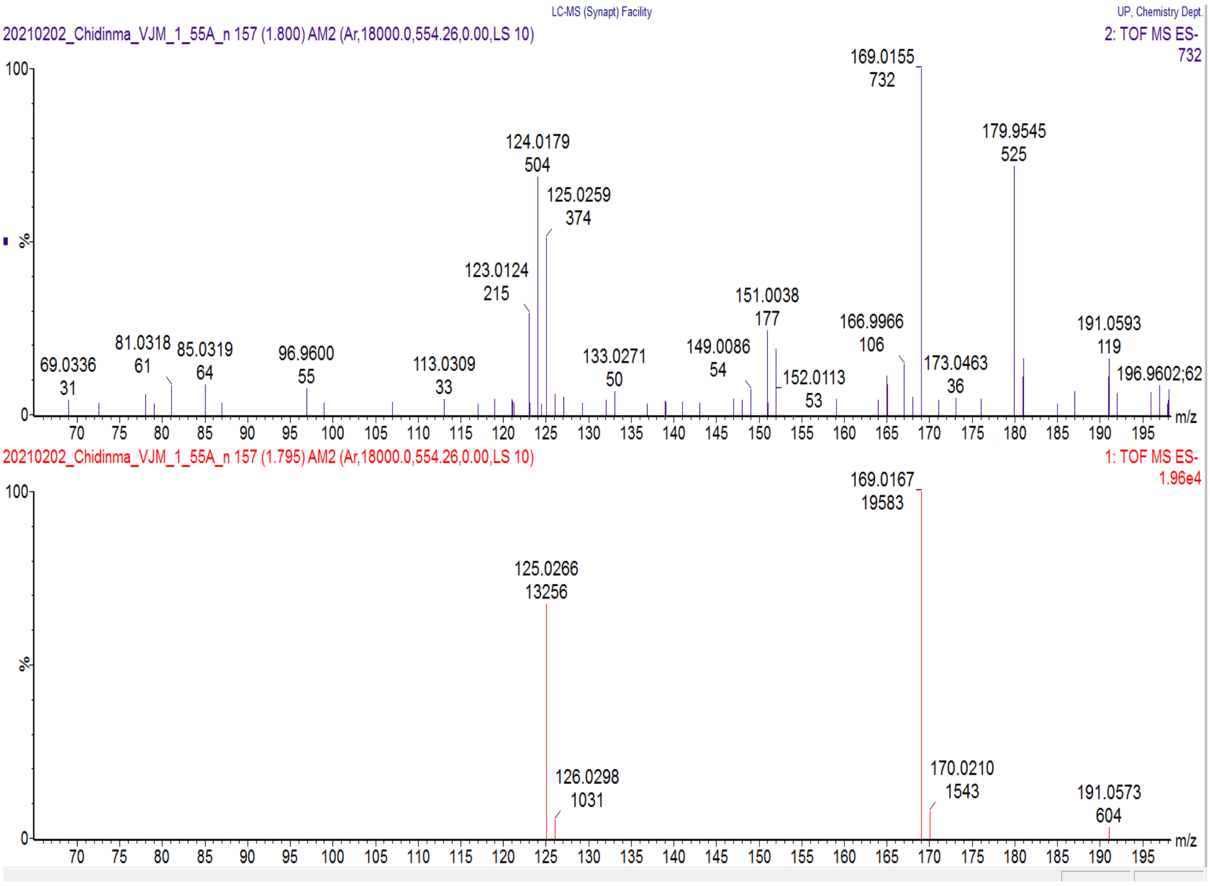

Fig.S9. MS fragmentation pattern of peak 2 overlaid with MSMS fragmentation pattern of peak 2

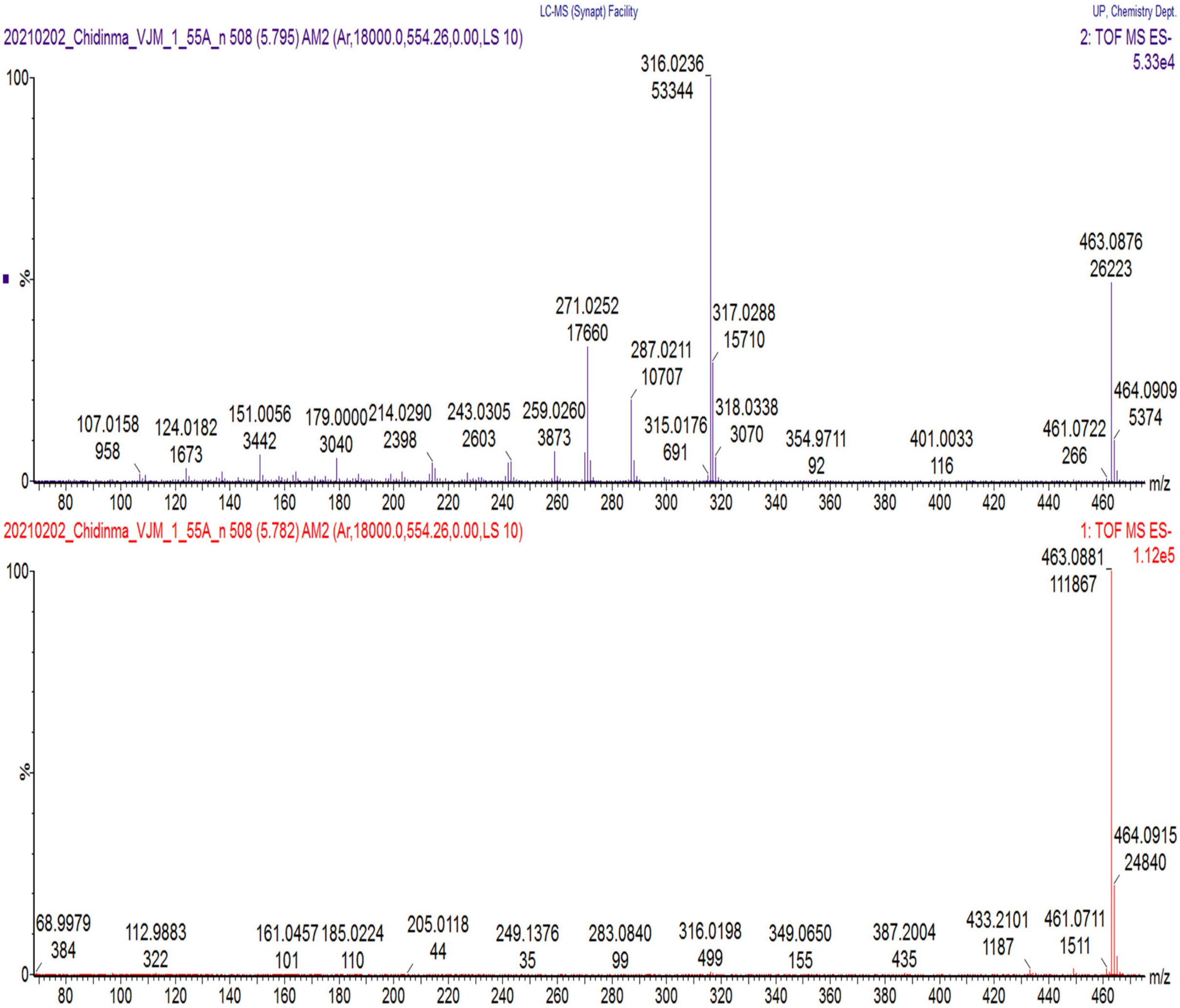

Fig.S10. MS fragmentation pattern of peak 3 overlaid with MSMS fragmentation pattern of peak 3

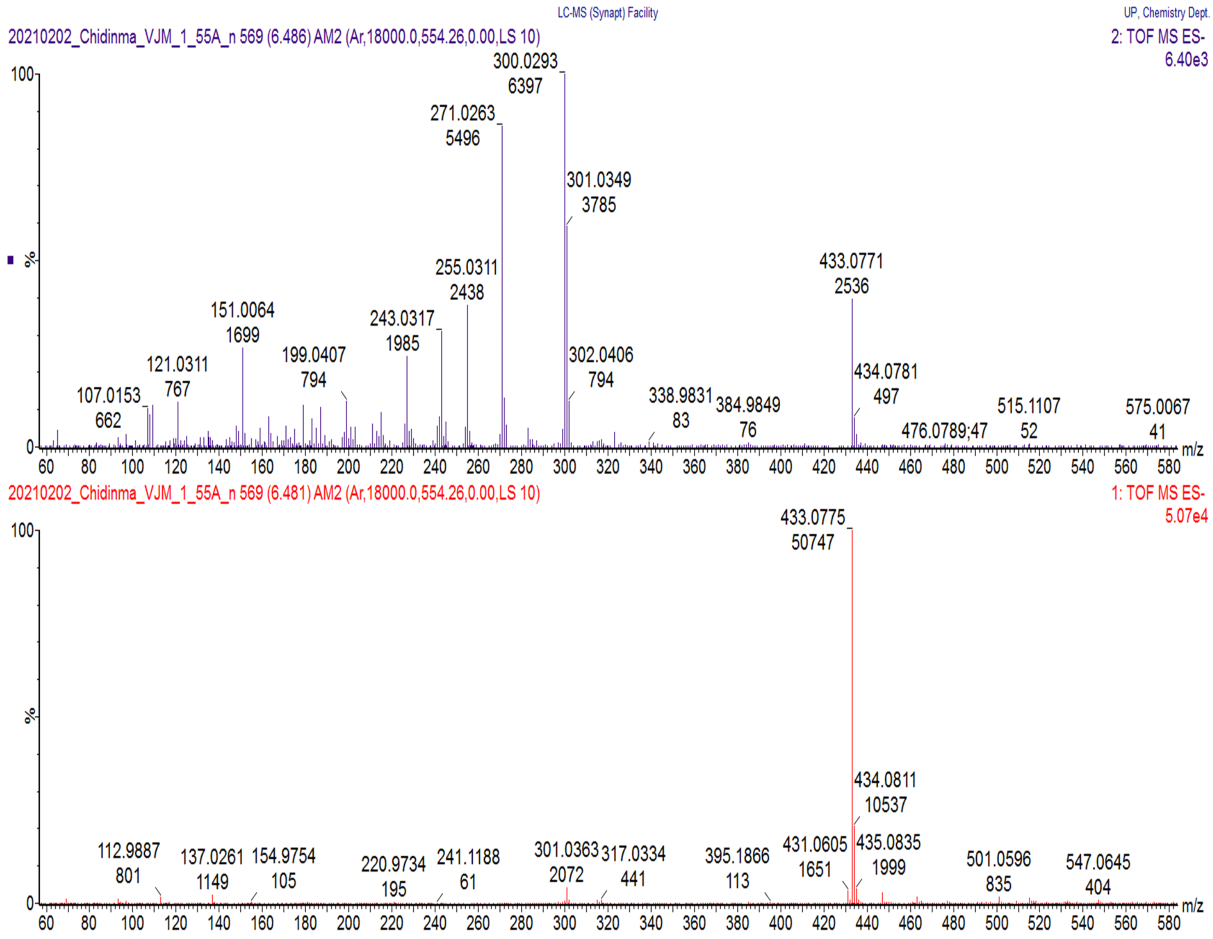

Fig.S11. MS fragmentation pattern of peak 4 overlaid with MSMS fragmentation pattern of peak 4

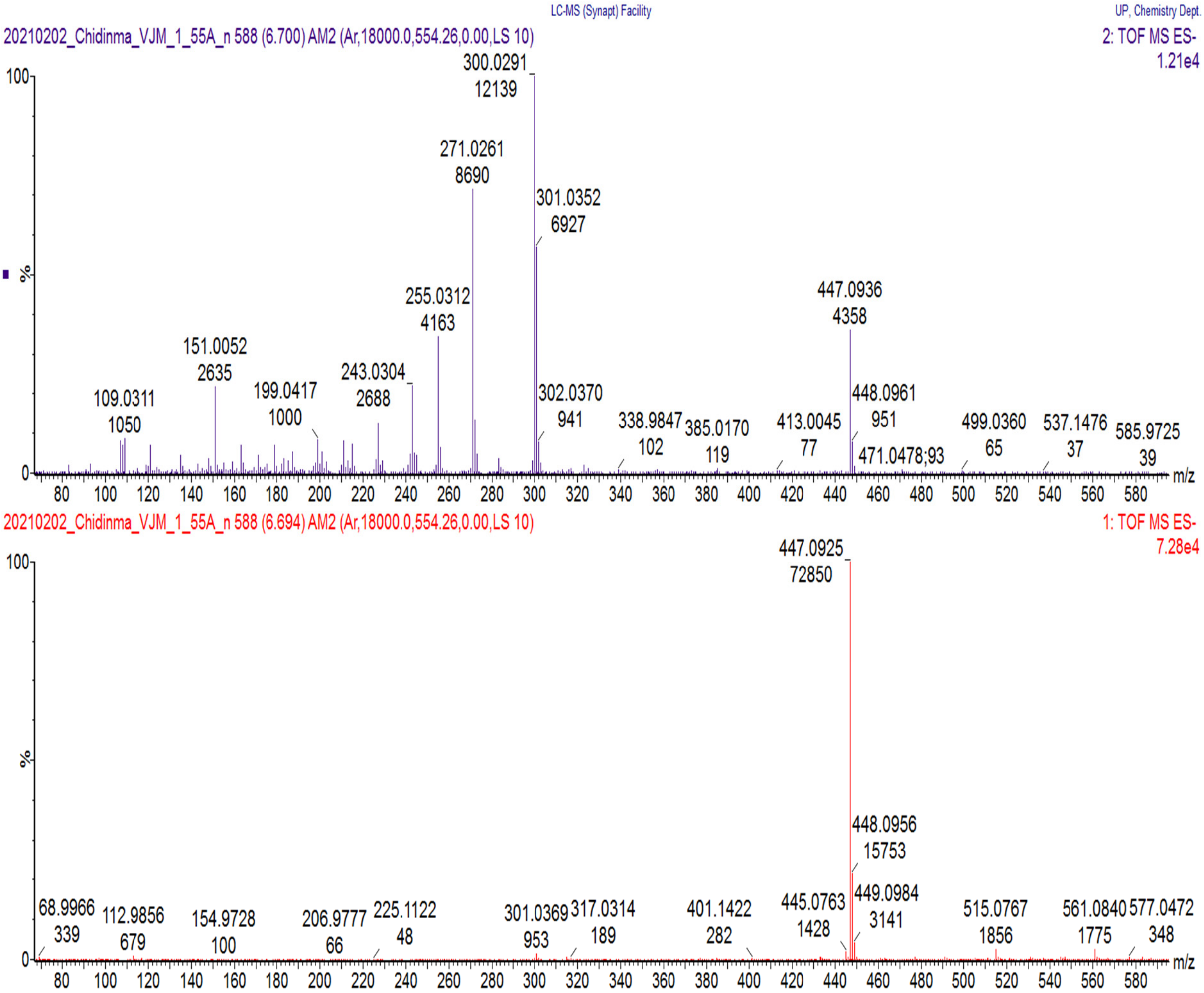

Fig.S12. MS fragmentation pattern of peak 5 overlaid with MSMS fragmentation pattern of peak 5

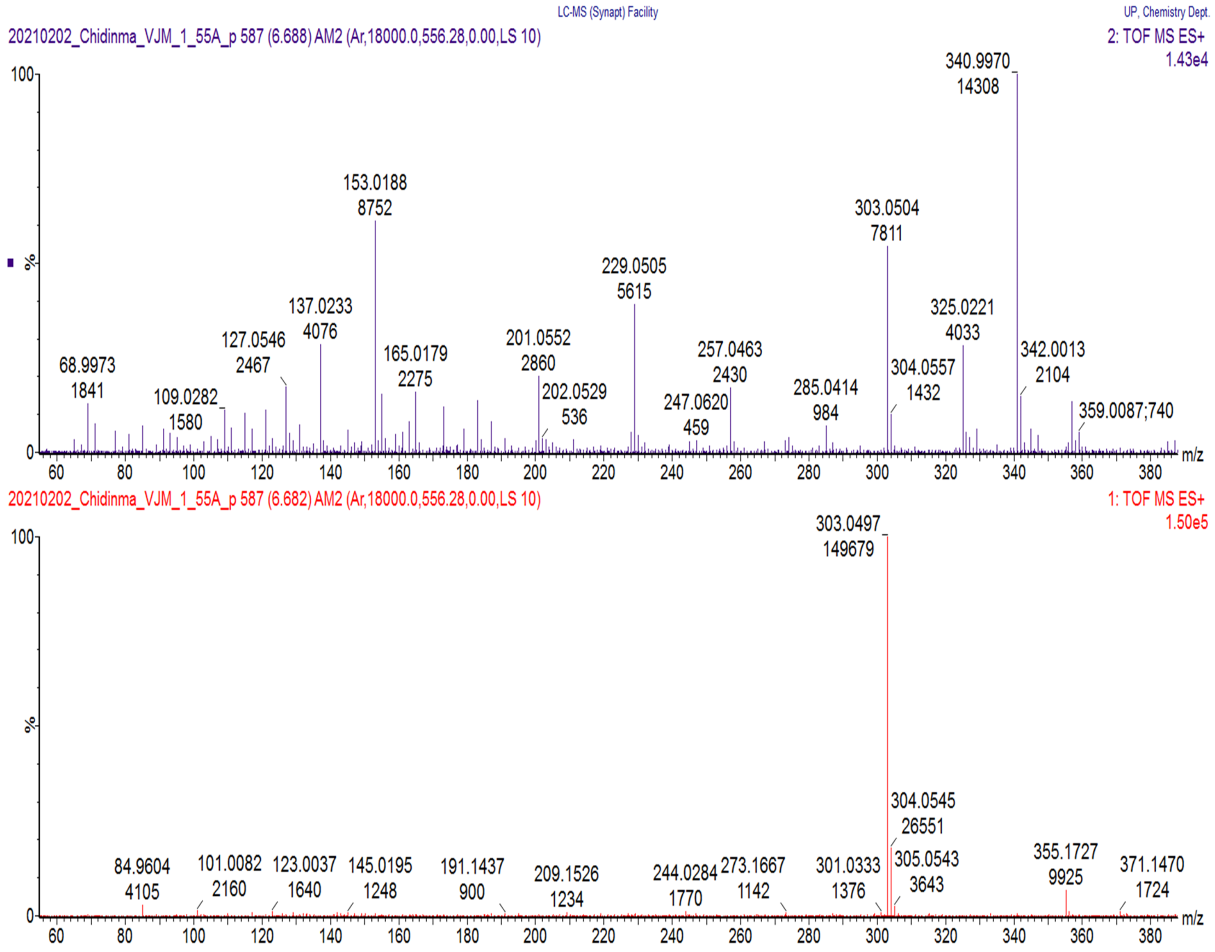

Fig.S13. MS fragmentation pattern of peak 6 overlaid with MSMS fragmentation pattern of peak 6

Isolation of the chemical constituents

Compound **1** was isolated as a light-yellow solid. It had a molecular formula of $C_{15}H_{11}O_8$ as deduced from its monoprotonated molecular ion at *m/z* 319.0443 (calcd for $[M+H]^+$ *m/z* 319.0454) based on the QTOF mass spectrum with eleven degrees of unsaturation. The ^{13}C NMR spectroscopic data showed it to have a flavonol skeleton confirming the presence of fifteen carbon atoms, of which four are aromatic methine groups and eleven quartenary carbons (one carbonyl, six O-bearing and four aliphatic) as deduced from its DEPT 135 spectrum. The ^1H NMR spectrum showed the presence of four aromatic protons; two protons resonating at δ_H 6.22 (1H, d, J = 2.09 Hz, H-6) and δ_H 6.39 (1H, d, J = 2.17 Hz, H-8) consistent with the meta coupled protons at H-6 and H-8 positions on the A-ring and a signal at δ_H 6.97 (2H, s, H-2', H-6') indicating presence of two protons at 2'and 6' positions appearing as a singlet due to their para substituted ring B which led to their symmetrical pattern. This indicated that the molecule was a flavonoid, which matched myricetin, previously isolated from black currant [38].

Compound **2** was isolated as a light-yellow solid. It had a molecular formula of $C_{21}H_{19}O_{14}$ as deduced from its monoprotonated molecular ion at *m/z* 495.0886 (calcd for $[M+H]^+$ *m/z* 495.0775) based on the QTOF mass spectrum with thirteen degrees of unsaturation. The ^{13}C-NMR spectrum showed the presence of nine methine groups (four aromatic methine and five glucuronic acid methine) and twelve quartenary carbons (one carbonyl, one carboxylic acid, six O-bearing, four aliphatic) as deduced from its DEPT 135 spectrum. The ^1H NMR spectrum showed the presence of four aromatic protons; two protons resonating at δ_H 6.21 (1H, d, J =

2.05 Hz, H-6) and δ_H 6.39 (1H, d, J = 2.00 Hz, H-8) consistent with the meta coupled protons at H-6 and H-8 positions on the A-ring and a signal at δ_H 7.29 (2H, s, H-2', H-6') indicating presence of two protons at 2'and 6' positions appearing as a singlet due to their para substituted ring B which led to their symmetrical pattern. The proposed structure was confirmed by the comparative analysis of the reported myricetin-3-O-β-D-glucuronide to that of compound **2** [39].

Compound **3** was isolated as a light-yellow solid. It had a molecular formula of $C_{21}H_{18}O_{13}$ as deduced from its monoprotonated molecular ion at *m/z* 479.0952 (calcd for $[M+H]^+$ *m/z* 479.0826) based on the QTOF mass spectrum with thirteen degrees of unsaturation. The ^{13}C NMR spectrum showed the presence of ten methine groups (five aromatic methine and five glucuronic acid methine) and eleven quartenary carbons (one carbonyl, one carboxylic acid, five O-bearing and four aliphatic) as deduced from its DEPT 135 spectrum. The ^1H NMR spectrum showed signals corresponding to five aromatic protons (δ_H 6.10-7.78). Two proton signals at δ_H 6.10 (1H, d, J = 2.05 Hz, H-6) and 6.30 (1H, d, J = 2.05 Hz, H-8) attributable to the A ring of quercetin which were assigned to H-6 and H-8 positions respectively. Additionally, three proton signals at δ_H 7.78 (1H, bs, H-2'), 6.75 (1H, d, J = 8.48 Hz, H-5') and 7.44 (1H, dd, J = 2.15 and 2.18 Hz, H-6') were assigned to the B ring of quercetin as H-2', H-5' and H-6' respectively. These proton signals confirmed that the structure of the aglycone moiety was quercetin. The proposed structure was finally confirmed by the comparative analysis of the reported quercetin-3-O-β-D-glucuronide to that of compound **3** [39].
